# Aging modifies the effect of cardiac output on middle cerebral artery blood flow velocity

**DOI:** 10.14814/phy2.13361

**Published:** 2017-09-15

**Authors:** Anne‐Sophie G. T. Bronzwaer, Jasper Verbree, Wim J. Stok, Mat J. A. P. Daemen, Mark A. van Buchem, Matthias J. P. van Osch, Johannes J. van Lieshout

**Affiliations:** ^1^ Department of Internal Medicine Academic Medical Center University of Amsterdam Amsterdam The Netherlands; ^2^ Laboratory for Clinical Cardiovascular Physiology Center for Heart Failure Research Academic Medical Center University of Amsterdam Amsterdam The Netherlands; ^3^ Department of Radiology Leiden University Medical Center Leiden The Netherlands; ^4^ Department of Medical Biology Academic Medical Center University of Amsterdam Amsterdam The Netherlands; ^5^ Department of Pathology Academic Medical Center University of Amsterdam Amsterdam the Netherlands; ^6^ MRC/Arthritis Research UK Centre for Musculoskeletal Ageing Research School of Life Sciences University of Nottingham Medical School Queen's Medical Centre Nottingham UK

**Keywords:** Aging, cerebral autoregulation, cerebral blood flow, hemodynamics, physiology, transcranial Doppler

## Abstract

An association between cerebral blood flow (CBF) and cardiac output (CO) has been established in young healthy subjects. As of yet it is unclear how this association evolves over the life span. To that purpose, we continuously recorded mean arterial pressure (MAP; finger plethysmography), CO (pulse contour; CO‐trek), mean blood flow velocity in the middle cerebral artery (MCAV; transcranial Doppler ultrasonography), and end‐tidal CO_2_ partial pressure (PetCO_2_) in healthy young (19–27 years), middle‐aged (51–61 years), and elderly subjects (70–79 years). Decreases and increases in CO were accomplished using lower body negative pressure and dynamic handgrip exercise, respectively. Aging in itself did not alter dynamic cerebral autoregulation or cerebrovascular CO_2_ reactivity. A linear relation between changes in CO and MCAV_mean_ was observed in middle‐aged (*P* < 0.01) and elderly (*P* = 0.04) subjects but not in young (*P* = 0.45) subjects, taking concurrent changes in MAP and PetCO_2_ into account. These data imply that with aging, brain perfusion becomes increasingly dependent on CO.

## Introduction

The brain is a highly metabolic active organ and even short‐lasting discontinuation of its blood supply has deleterious functional effects (Heiss and Rosner 1983). Cerebral blood flow (CBF) is tightly regulated by control systems including the cerebral autoregulation, cerebrovascular CO_2_ reactivity, and neurovascular coupling (Willie et al. 2014). The traditional concept of autoregulation predicts constancy of CBF as long as fluctuations in blood pressure remain limited to the so‐called autoregulatory range and arterial CO_2_ tension remains stable. However, a separate influence of cardiac output (CO) on CBF beyond blood pressure has been considered both in health and disease (Ide et al. 1998, 1999; Meng et al. 2015). Acute deliberate alterations in CO by manipulating cardiac preload in young healthy volunteers, for instance, a decrease by lower body negative pressure (Levine et al. 1994; Brown et al. 2003; Ogoh et al. 2005; Ogawa et al. 2007) and standing up (Van Lieshout et al. 2001), or an increase by infusion of albumin or saline (Ogoh et al. 2005; Ogawa et al. 2007), lead to noticeable changes in middle cerebral artery blood flow velocity (MCAV) as measured by transcranial Doppler (TCD). Also, compromised cardiac function, as in patients with heart failure, is associated with a low CBF (Paulson et al. 1984; Rajagopalan et al. 1984; Paulson et al. 1986; Gruhn et al. 2001; Choi et al. 2006; Vogels et al. 2008; Loncar et al. 2011). This reduction in CBF seems reversible by interventions that improve CO including cardiac transplantation (Gruhn et al. 2001; Choi et al. 2006; Massaro et al. 2006) and cardiac resynchronization therapy (van Bommel et al. 2010; Ozdemir et al. 2013). As of yet the importance of CO for CBF over the life span has not been defined.

Aging in itself is associated with structural and functional alterations of the heart and arterial blood vessels, such as an increase in left ventricular wall thickness, alterations in diastolic filling pattern and a reduction in arterial compliance leading to arterial wall stiffening (Wei 1992; Cheitlin 2003; Lakatta 2003). In addition, in the elderly, peak CO declines in response to exercise with blunting of the heart rate (HR) response which relates to aging on cardiovascular reserve capacity (Shannon et al. 1991; Folkow and Svanborg 1993; Fleg et al. 1995; van Hoeyweghen et al. 2001). Aging is also associated with a decline in CBF, resting cerebral metabolism, and weight of the brain (Spann and Dustmann 1965; Shaw et al. 1984; Chen et al. 2011). Specifically, with aging, the capability to increase cerebrovascular conductance in response to brain activation by exercise becomes reduced (Fisher et al. 2013).

We questioned whether aging affects the relationship between MCAV and CO. We studied the effect of varying CO from lower to higher values by, respectively, lower body negative pressure (LBNP) and dynamic handgrip (HG) exercise on MCAV in healthy young, middle‐aged, and elderly subjects.

## Methods

### Ethical approval

The study protocol was approved by the Medical Ethics Committee from the Academic Medical Center (Amsterdam, the Netherlands) and performed in accordance with the Declaration of Helsinki. Written informed consent was obtained from all participants prior to the experiments.

### Subjects

Eighteen young (19–27 years; 9 females), 20 middle‐aged (51–61 years; 9 females), and 19 elderly (70–79 years; 6 females) healthy subjects participated in this study. All subjects underwent a medical screening prior to the experiment including a medical interview, fasting blood sampling (including plasma hemoglobin, hematocrit, HbA1C, creatinine, glucose, total cholesterol, HDL cholesterol, and LDL cholesterol), urine sampling (microalbumin), and an electrocardiogram (ECG). Subjects were excluded from participation in case of a medical history of cardiovascular disease, hypertension, diabetes mellitus, and/or neurological disease; use of vasoactive medication; abnormal ECG and/or laboratory results; and/or smoking or having smoked within 10 years. Subjects abstained from heavy exercise and caffeinated beverages for at least 5 h prior to the experiment.

### Experimental protocol

Measurements were performed in a quiet and temperature controlled (20–22°C) room with the subjects supine. To induce changes in CO, two challenges were performed. The protocol started with a LBNP session to reduce CO, to be followed by a dynamic handgrip (HG) exercise session to increase CO. Each session included 5 min of rest followed by a 5 min trial of either LBNP or HG exercise. This was repeated three times during each session. During the experiment, subjects were coached to breathe normally. After instrumentation, a cerebral vasomotor reactivity test was performed.

#### Lower body negative pressure

The lower body of the subject was positioned inside the LBNP box (Dr. Kaiser Medizintechnik, Bad Hersfeld, Germany) and sealed at the level of the iliac crest (Goswami et al. 2009). The subatmospheric pressure inside the box was set to −50 mmHg and established within 10 sec. The box was equipped with a saddle to avoid leg muscle pump activation during the application of subatmospheric pressure. LBNP was terminated upon request by the volunteer or in case of (pre‐)syncopal symptoms including sweating, light headedness, nausea, or blurred vision, and/or signs meeting one or more of the following criteria: systolic arterial pressure (SAP) below 80 mmHg or rapid drop (SAP by ≥20 mmHg/min, diastolic [DAP] by ≥10 mmHg/min), drop in HR by ≥15 bpm.

#### Dynamic handgrip exercise

At the start of the exercise session, maximum voluntary contraction (MVC) was assessed by squeezing the dynamometer (gripforce 500N, Curdes, Philadelphia PA, USA) to the maximum extent possible. Dynamic HG exercise consisted of repeated 2 sec hand contractions alternated with 2 sec of relaxation. Hand contractions started at 80% of MVC during the first minute, and force was then lowered to 60% of MVC for the remaining 4 min. The applied pressure was displayed as relative force on a screen, providing the subject with real‐time visual feedback.

#### Cerebral vasomotor reactivity

Cerebrovascular CO_2_ responsiveness was expressed as the change in MCAV for a given change in PetCO_2_ (Tominaga et al. 1976). A wide range of PetCO_2_ was established by, respectively, inhaling a gas mixture containing 5% CO_2_ and 95% O_2_ through a mouthpiece for 2 min, followed by 2 min of breathing room air and hyperventilating for approximately 1.5 min.

### Measurements

Continuous blood pressure (BP) was measured noninvasively by finger plethysmography with the cuff placed around the middle phalanx of the nondominant hand placed at heart level (Nexfin, Edwards Lifesciences BMEYE, the Netherlands). Left ventricular stroke volume (SV) was estimated beat by beat by pulse contour (Nexfin CO‐trek, Edwards Lifesciences BMEYE, Amsterdam, the Netherlands) and by inert gas rebreathing (Innocor, Innovision A/S, Odense, Denmark) (Gabrielsen et al. 2002; Bartels et al. 2011). CO was stroke volume (SV) times heart rate (HR). Total peripheral resistance (TPR) was the ratio of mean arterial pressure (BP_mean_) and CO. End‐tidal CO_2_ partial pressure (PetCO_2_) was monitored through a nasal cannula connected to a sampling capnograph (Datex Normocap 200, Helsinki, Finland).

Changes in MCAV were followed in the proximal segment of the middle cerebral artery (MCA) by transcranial Doppler ultrasonography (TCD; DWL Multidop X4, Sipplingen, Germany). The left MCA was insonated through the temporal window just above the zygomatic arch at a depth of 40–60 mm with a pulsed 2 MHz probe. Once the optimal signal‐to‐noise ratio was obtained, the probe was immobilized by a head band.

### Data analysis

Signals were inspected for artifacts and analyzed offline. During the last 3 min of, respectively, resting, LBNP, and handgrip exercise periods (average of three trials), the relation between CO and MCAV_mean_ was assessed. Relative changes in CO and MCAV_mean_ were calculated by: Δ% = (*B*−*A*)/*A* ×100%, where *B* is the mean value during LBNP (or handgrip exercise) and *A* the mean value during the baseline period prior to either LBNP (or handgrip exercise). Cerebrovascular CO_2_ responsiveness was quantified by taking the last 30 sec of the three studied levels of PetCO_2_ (hypercapnia, normocapnia, and hypocapnia) assuring that the analysis was performed under steady‐state conditions.

The cerebrovascular CO_2_ responsiveness was expressed as relative change in MCAV_mean_ in response to absolute change in PetCO_2_. Cerebrovascular resistance index (CVRi) was calculated as the ratio of BP_mean_ and MCAV_mean_. Dynamic cerebral autoregulation (CA) was quantified in the frequency domain as the counter‐regulatory capacity to maintain MCAV during spontaneous oscillations in BP (Panerai et al. 1998). The last 3 min of the baseline period prior to LBNP were used for analysis of dynamic CA. Beat‐to‐beat BP_mean_ and mean MCAV_mean_ data were spline interpolated and resampled at 4 Hz. Power spectra were estimated by transforming the time series with discrete Fourier transformation to the frequency domain. With cross‐spectral density analysis, transfer function phase shift and gain were derived in the low‐frequency range (LF; 0.07–0.15 Hz). The transfer function gain was normalized for BP_mean_ and MCAV_mean_ to account for the intersubject variability and expressed as % change in cm·s^−1^ per % change in mmHg (Panerai et al. 1999; Immink et al. 2004). Phase was defined positive where MCAV_mean_ leads BP_mean_. The coherence function reflects the fraction of output power (MCAV_mean_) that can be linearly related to the input power (BP_mean_) at the LF range. A coherence above 0.5 between BP and MCAV recordings was considered to provide a reliable estimate of the transfer function variables.

### Statistical analysis

Variables are presented as mean ± SD. The effect of LBNP and HG exercise on measured parameters was assessed using a paired two‐tailed Student's *t*‐test (Sigmaplot 11.0, Systat Software Inc., USA). Differences between age groups were assessed using one‐way ANOVA followed by a Tukey's post hoc test (Sigmaplot 11.0, Systat Software Inc., USA). The relation between ΔCO on ΔMCAV_mean_ was evaluated by a linear mixed regression model (fitlme function, Matlab 2016a Statistics toolbox 9.0.0.341360) using maximum likelihood estimation. A model with random slope and intercept was used while grouping the measurements by subject to account for repeated measurements (LBNP and HG). The effect of age on the CO–MCAV_mean_ relationship was investigated by adding age group (young, middle‐aged, and elderly subjects) and the interaction with CO (age group × CO) as fixed effects. This model was designated as the “basic model.” The basic model was extended into an “extended model” with ΔBP_mean_ and ΔPetCO_2_ as additional fixed effects as these parameters were expected to also affect mean MCAV_mean_. The extended model was used to evaluate the effect of CO on MCAV_mean_, while accounting for concurrent changes in BP_mean_ and PetCO_2_. The difference in CO–MCAV_mean_ regression slopes across age groups was assessed using post hoc *F* tests. The normal distribution of the residuals of the final model was visually confirmed. The probability level for statistical significance was set equal to *P* = 0.05.

## Results

From the 57 healthy subjects included in this study, data from 17 subjects were excluded from analysis based on medical screening (2 young, 1 middle aged, and 4 elderly), insufficient quality of Nexfin and/or TCD signals (1 young, 4 middle aged, and 4 elderly), or refusal of further participation after medical screening by the subject (1 middle aged), leaving data from 40 subjects available for analysis. Baseline subject characteristics are provided in Table 1.

**Table 1:** Subject characteristics

	Young	Middle aged	Old
*n* (male/female)	15 (8/7)	14 (8/6)	11 (10/1)
Mean age (years)	23 ± 3	56 ± 4	72 ± 3
Body mass (kg/m^2^)	22 ± 2	24 ± 3	25 ± 2
BP (mmHg)			
Systolic	123 ± 12	136 ± 14^a^	148 ± 17^a^
Mean	90 ± 9	96 ± 8	101 ± 11^a^
Diastolic	72 ± 7	73 ± 5	74 ± 8
HR (beats·min^−1^)	58 ± 8	55 ± 5	60 ± 6
CO_CO‐trek_ (L·min^−1^)	6.4 ± 0.9	4.9 ± 0.7^a^	4.7 ± 0.8^a^
CO_rebreathing_ (L·min^−1^)	7.9 ± 2.2	5.7 ± 1.0^a^	5.8 ± 1.0^a^
Mean MCAV (cm·s^−1^)	72 ± 14	59 ± 8^a^	57 ± 15^a^
PetCO_2_ (mmHg)	41 ± 4	41 ± 5	38 ± 6

Data are presented as mean ± SD. BP, blood pressure; HR, heart rate; CO, cardiac output; MCAV, middle cerebral artery blood flow velocity; PetCO_2_, end‐tidal CO_2_ partial pressure.

a *P* < 0.05 versus young.

### Baseline

Baseline systolic BP was higher and CO (assessed by either CO‐trek or rebreathing) and MCAV_mean_ were lower in the middle‐aged and elderly subjects compared to the young with no change between middle‐aged and elderly subjects (Table 1). Furthermore, BP_mean_ was higher in the group of elderly subjects compared to the young but not to the middle‐aged subjects. Baseline HR and PetCO_2_ did not differ between groups. BP_mean_ was positively correlated with age (*r* = 0.43, *P* = 0.006), whereas CO (*r* = −0.72. *P* < 0.001) and MCAV_mean_ (*r* = −0.49, *P* = 0.001) were negatively correlated with age.

### CO and MCAV_mean_

**Figure 1:**
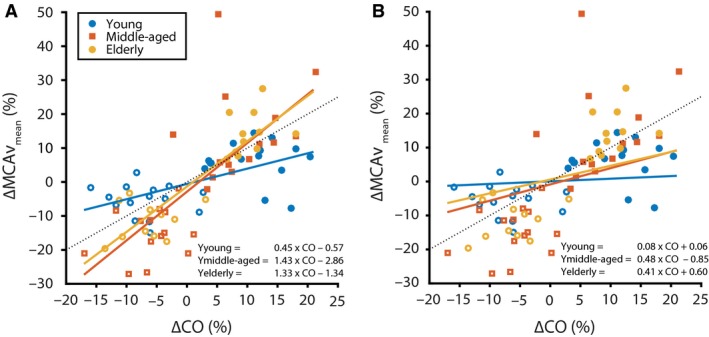
Effect of age group on the CO–MCAV_mean_ relation for (A) the basic model and (B) the extended model accounting for concurrent changes in MAP and PetCO_2_. Dashed line represents line of unity. The equations for the regression line are given for the three age groups. The presented values are relative to the baseline condition.

**Figure 2:**
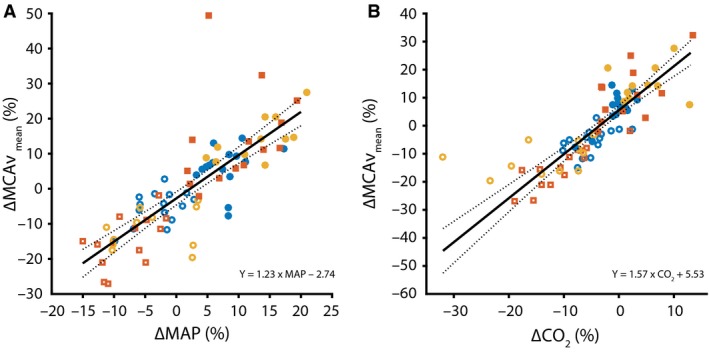
Relation between ∆% MCAV_mean_ and ∆% (A) MAP (*n* = 80) and (B) PetCO_2_ (*n* = 77). Same color legend as in Figure 1. The black line indicates the regression slope (solid) with 95% confidence intervals (dashed). The equations for the regression line, using data from all age groups, are depicted. The presented values are relative to the baseline condition.

The hemodynamic responses to LBNP and HG exercise are presented in Table 2. Figure 1A illustrates the linear relation between ΔCO and ΔMCAV_mean_ as obtained from both the basic model with a significant effect of age on this relationship (*P* < 0.01). Figure 2 depicts the linear relation between ΔMCAV_mean_ and ΔBP (*P* < 0.001) and between ΔMCAV_mean_ and ΔPetCO_2_ (*P* < 0.001), which was significant for all age groups. In the extended model (Fig. 1B), accounting for concurrent changes in BP_mean_ and PetCO_2_, the linear relation between ΔCO on ΔMCAV_mean_ was no longer present in the young subjects (*P* = 0.45), but remained significant in the middle‐aged (*P *< 0.04) and elderly (*P* = 0.04) groups. Post hoc comparison indicated no statistical difference in slope between the middle‐aged and the young group (*F*(1, 69) = 2.57, *P* = 0.11) or between the elderly and young group (*F*(1, 69) = 1.37, *P* = 0.25). The random effects (slope and intercept) controlling for the within‐subject variance were not significant, and removing them did not improve the model (*P* = 0.83).

**Table 2:** Hemodynamic response to lower body negative pressure (LBNP) and handgrip exercise (HG)

	LBNP		HG	
	Baseline	∆ (%)	Baseline	∆ (%)
BP_mean_ (mmHg)				
Young	90 ± 9	−3 ± 4^a^	94 ± 9	8 ± 3^a^
Middle aged	96 ± 8	−8 ± 4^a^^,^ b	98 ± 7	10 ± 6^a^
Old	101 ± 11^b^	−4 ± 6	103 ± 12^b^	13 ± 5^a^^,^ b
HR (bpm)				
Young	58 ± 8	31 ± 18^a^	61 ± 7	12 ± 6^a^
Middle aged	55 ± 5	28 ± 15^a^	57 ± 5	10 ± 5^a^
Old	60 ± 6	21 ± 13^a^	61 ± 7	10 ± 3^a^
CO (L·min^−1^)				
Young	6.4 ± 0.9	−7 ± 5^a^	6.9 ± 0.8	11 ± 6^a^
Middle aged	4.9 ± 0.7^b^	−6 ± 5^a^	5.1 ± 0.8^b^	9 ± 6^a^
Old	4.7 ± 0.8^b^	−6 ± 5^a^	4.8 ± 0.8^b^	10 ± 3^a^
TPR (dyn·sec·cm^−5^)				
Young	1142 ± 114	4 ± 5^a^	1114 ± 112	−3 ± 5
Middle aged	1601 ± 211^b^	−2 ± 7	1543 ± 208^b^	1 ± 5
Old	1767 ± 350^b^	2 ± 10	1747 ± 336^b^	3 ± 5^b^
MCAV_mean_ (cm·sec^−1^)				
Young	72 ± 14	−5 ± 5^a^	72 ± 14	6 ± 6^a^
Middle aged	59 ± 8^b^	−15 ± 7^a^^,^ b	57 ± 8^b^	14 ± 14^a^
Old	57 ± 15^b^	−11 ± 6^a^^,^ b	55 ± 14^b^	14 ± 6^a^
CVRi (mmHg·cm^−1^·sec)				
Young	1.30 ± 0.20	3 ± 5	1.35 ± 0.23	2 ± 6
Middle aged	1.66 ± 0.26^b^	9 ± 9^a^	1.73 ± 0.21^b^	−3 ± 10
Old	1.89 ± 0.57^b^	9 ± 9^a^	1.99 ± 0.56^b^	−1 ± 4
PetCO_2_ (mmHg)				
Young	41 ± 4	−5 ± 4^a^	40 ± 3	−1 ± 3
Middle aged	41 ± 5	−10 ± 6^a^^,^ b	40 ± 2	2 ± 5
Old	38 ± 6	−14 ± 9^a^^,^ b	37 ± 4^b^^,^ c	5 ± 5^a^^,^ b

Data are presented as mean ± SD. TPR, total peripheral resistance; CVRi, cerebrovascular resistance index; BP, blood pressure; HR, heart rate; CO, cardiac output; MCAV, middle cerebral artery blood flow velocity; PetCO_2_, end‐tidal CO_2_ partial pressure.

a *P *< 0.05 versus baseline.

b *P *< 0.05 versus young.

c *P *< 0.05 versus middle aged.

### Dynamic cerebral autoregulation

There was no difference in LF BP_mean_ or MCAV_mean_ power between age groups. The BP_mean_ to MCAV_mean_ phase lead and normalized gain were comparable among groups (Table 3).

**Table 3:** Transfer function gain, phase and coherence

	Young	Middle aged	Elderly
Mean BP_power_, mmHg^2^·Hz^−1^	2.9 ± 2.0	3.1 ± 2.1	3.2 ± 1.6
Mean MCAV_power_, (cm·sec^−1^)^2^·Hz^−1^	3.5 ± 2.4	2.0 ± 1.5	1.8 ± 1.0
Coherence, k	0.61 ± 0.16	0.57 ± 0.13	0.73 ± 0.08^a^
Phase (°)	43 ± 19	49 ± 27	41 ± 9
Gain, (cm·s^−1^)·mmHg^−1^	0.97 ± 0.28	0.65 ± 0.13^b^	0.68 ± 0.23^b^
Normalized gain, %.%^−1^	1.19 ± 0.97	1.14 ± 0.51	1.14 ± 0.63

Data are presented as mean ± SD. BP, blood pressure; MCAV, middle cerebral artery blood flow velocity.

a *P *< 0.05 versus middle aged.

b *P *< 0.05 versus young.

### Cerebral vasomotor reactivity

**Figure 3:**
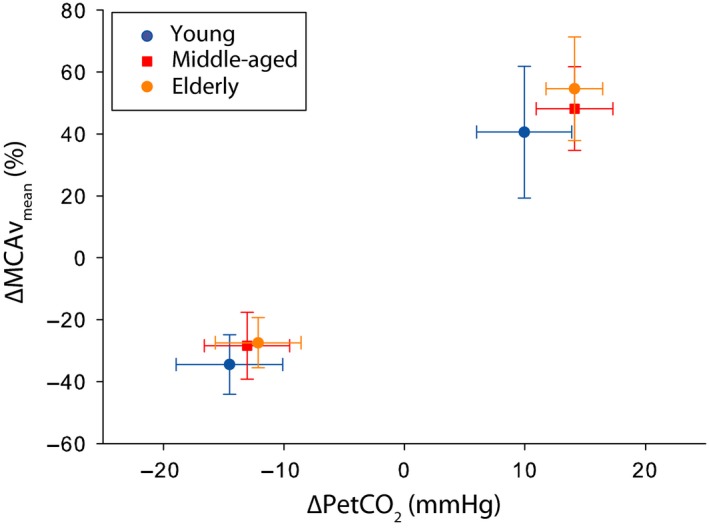
Percentage change in MCAV_mean_ per mmHg change in PetCO_2_ in response to hyperventilation and inhalation of 5% CO_2_.

Baseline values of MCAV_mean_ were lower in the middle‐aged and elderly groups compared to the young subjects, whereas baseline PetCO_2_ and BP_mean_ did not differ. The cerebrovascular CO_2_ responsiveness was comparable for all age groups (*P* = 0.341; Fig. 3 and Table 4).

**Table 4:** Cerebral vasomotor reactivity

	Young (*n* = 12)	Middle aged (*n* = 14)	Elderly (*n* = 11)
Baseline (breathing room air)			
Mean BP (mmHg)	92 ± 13	94 ± 10	100 ± 11
Mean MCAV (cm·s^−1^)	69 ± 16	55 ± 12^b^	54 ± 14^b^
PetCO_2_ (mmHg)	40 ± 4	40 ± 4	37 ± 5
Hyperventilation			
Mean BP (mmHg)	93 ± 14	96 ± 11	105 ± 10^a^
Mean MCAV (cm·s^−1^)	45 ± 10^a^	38 ± 6^a^	39 ± 8^a^
PetCO_2_ (mmHg)	26 ± 3^a^	26 ± 3^a^	24 ± 4^a^
Inhalation of 5% CO_2_			
Mean BP (mmHg)	96 ± 14^a^	105 ± 13^a^	114 ± 12^a^^,^ b
Mean MCAV (cm·sec^−1^)	97 ± 24^a^	80 ± 14^a^	83 ± 23^a^
PetCO_2_ (mmHg)	50 ± 5^a^	54 ± 4^a^	51 ± 4^a^
Full range PetCO_2_			
∆ Mean MCAV/∆ PetCO_2_ (%·mmHg^−1^)	3.1 ± 0.7	2.8 ± 0.4	3.1 ± 0.7

Data are presented as mean ± SD. BP, blood pressure; HR, heart rate; CO, cardiac output; MCAV, middle cerebral artery blood flow velocity; PetCO_2_, end‐tidal CO_2_ partial pressure.

a *P *< 0.05 versus baseline.

b *P *< 0.05 versus young.

## Discussion

The findings of the present study provide new information regarding the influence of aging on the relationship between CBF and CO. Specifically, acute alterations in CO related to concomitant changes in MCAV_mean_ in healthy middle‐aged and elderly but not in young subjects. This observation suggests that with aging, brain perfusion becomes increasingly dependent on CO.

A relationship between CO and MCAV has previously been demonstrated in young healthy volunteers when central blood volume, and consequently CO, was acutely decreased (Levine et al. 1994; Van Lieshout et al. 2001; Brown et al. 2003; Ogoh et al. 2005; Ogawa et al. 2007), respectively, increased (Van Lieshout et al. 2001; Ogoh et al. 2005; Ogawa et al. 2007), with an average of 0.35% change in MCAV_mean_ per 1% change in CO (Meng et al. 2015). In those studies, an attempt was made to minimize changes in BP and PetCO_2_, but the regression to MCAV_mean_ with CO as a single input variable (Meng et al. 2015) (similar to our basic model) did not account for small but inevitable changes in these variables. In the present study, the basic model demonstrated a comparable relationship in a similar age group (*young*), that is, a 0.45% change in MCAV_mean_ in response to 1% change in CO. In contrast, according to our extended model with BP_mean_ and PetCO_2_ added as additional input variables, a relation between CO and MCAV_mean_ was no longer present. These findings indicate that in young healthy subjects, mild alterations in CO have no effect on MCAV_mean_ when correcting for concurrent changes in BP and PetCO_2_. Importantly, in both middle‐aged and elderly healthy subjects, the CO–MCAV_mean_ relationship remained significant also when accounting for the separate contributions of BP_mean_ and PetCO_2_. Apparently, aging discloses a separate relationship between CO and MCAV_mean_ beyond BP, suggesting that when growing older, CBF becomes more dependent on acute alterations in CO. We consider that acute changes in CO resulting from, for instance, dehydration, blood loss, myocardial infarction, and arrhythmia, all clinical conditions that are not uncommon in the elderly, impact on brain perfusion.

How alterations in CO relate to MCAV_mean_ in the middle‐aged and elderly but not in young healthy subjects is unknown. With aging the cerebral blood vessel wall properties change, with development of arteriosclerosis promoting arterial stiffness (Kalaria 1996; Fonck et al. 2009), while endothelial dysfunction and vessel wall smooth muscle cell degeneration facilitate sustained cerebral vasoconstriction (Iadecola 2004). In addition, aging is associated with enhanced sympathetic nervous system activity (Ng et al. 1993; Seals and Esler 2000) which, although still under debate, may also provoke vasoconstriction of small cerebral vessels (Levine et al. 1994). These findings are consistent with the larger cerebrovascular resistance observed in the present study comparing middle‐aged and elderly subjects to the young, both in the resting state as well as in response to sympathetic stimulation by central blood volume depletion. Considering an enhanced cerebrovascular resistance with increasing age, this may hinder the inflow of blood to the brain vasculature while growing older. On the other hand, advancing age inevitably leads to functional and structural alterations of the heart including left ventricular wall thickness, slowing of the left ventricular diastolic filling rate, a lower maximal HR, and a reduction of resting and maximal CO (Brandfonbrener et al. 1955; Lakatta 2003). Also, an altered response of cardiac volume to postural maneuvers is associated with aging. For instance, assumption of the sitting position from the supine position reduces end‐diastolic volume less in the older compared to young subjects (Fleg et al. 1995), with consequences for redistribution of CO (Ide et al. 1998; Fisher et al. 2013). Thus, the enhanced cerebrovascular resistance associated with aging hampers the inflow of blood to the brain vasculature, whereas aging‐related changes in cardiac structure and function may also reduce the amount of blood flowing toward the brain (Brandfonbrener et al. 1955; Lakatta 2003). Altogether, aging interferes with the functional capacity of both the heart and the brain to adapt to daily life environmental stress which can be considered a possible explanation for the effect of aging on the relationship between CO and CBF. Theoretically, malfunction of cerebral autoregulatory mechanisms could play a role in altered CBF control. However, the present study underscores that aging itself does neither affect dynamic autoregulatory capacity nor cerebral CO_2_ vascular responsiveness which conforms to previous research (Carey et al. 2000; Lipsitz et al. 2000; Oudegeest‐Sander et al. 2014). We therefore consider it unlikely that the aging effect on the CO–MCAV relationship observed in the present study is due to dysfunction of cerebrovascular autoregulatory integrity.

Potential limitations inherent to the study design should be considered. First, the challenges that were used to manipulate CO, especially the dynamic HG exercise test, are expected to also evoke a metabolic effect in the brain. Local cerebral metabolism is tightly coupled to local brain perfusion (Willie et al. 2014) and could have, in turn, contributed to the observed changes in MCAV_mean_ independently of CO. This so‐called neurovascular coupling is, however, unaffected by aging (Rosengarten et al. 2003) such that we consider it unlikely that it impacts on the present study outcome. Second, this study reports on the influence of acute alterations in CO on MCAV. From present findings, we cannot state whether these observations hold true for chronic changes in CO as well. Further studies in, for instance, patients with a chronically compromised cardiac function would add additional and interesting information on the CO–MCAV relationship over the life span. Third, it may be questioned whether PetCO_2_ tracks changes in arterial PCO_2_ when CO declines during LBNP. Generally, in a fixed body position, PetCO_2_ tracks changes in arterial CO_2_ partial pressure (PaCO_2_), and in healthy volunteers and patients, PetCO_2_ was comparable to PaCO_2_ across a wider range of hypocapnic and hypercapnic stimuli and breathing frequencies than created in the present study (Young et al. 1991; Ito et al. 2008) and has been applied since (Levine et al. 1994; Brothers et al. 2009). The relationship between CO and PetCO_2_ is linear (Weil et al. 1985) until CO declines by a very large reduction in central blood volume setting a limit to the supply of CO_2_ to the pulmonary vascular bed. When LBNP as a simulation of hemorrhage induces a reduction in CO, it becomes a rate‐limiting determinant of PetCO_2_ with a logarithmic CO–PetCO_2_ relationship (Ornato et al. 1990). The question then is whether the premise of a stable ventilation–perfusion (${\dot{V}}_{E}/\dot{Q}$) ratio is sufficiently met during LBNP to accept PetCO_2_ as a valid proxy for changes in arterial PCO_2_ and as input to the model used. Earlier we quantified the effect of the postural increase in ${\dot{V}}_{E}/\dot{Q}$ ratio on the arterial to end‐tidal CO_2_ gradient in response to active standing (Immink et al. 2006) and passive head‐up tilt (Immink et al. 2009). The ${\dot{V}}_{E}/\dot{Q}$ ratio increased by ~50% with on average a 1.8 mmHg overestimation of the postural reduction in PaCO_2_ by PetCO_2_ (4.8 ± 0.9 mmHg vs. 3.0 ± 1.1 mmHg). In contrast to the postural hydrostatic pressure gradient developing down the lungs with an influence on the distribution of blood over the lungs (Bjurstedt et al. 1962), supine LBNP plays only a minor role in affecting regional ventilatory parameters. This has been verified in studies on the effect of varying blood volume in the chest quantifying the posture‐related changing effects of gravity versus LBNP on the distribution of ventilation and aeration in the lungs (Frerichs et al. 2005; Bodenstein et al. 2014). Exposure to LBNP exerted a less appreciable effect on regional lung ventilation than the acute changes in gravity, and specifically in response to LBNP, the regional tidal volumes in the ventral and dorsal regions did not significantly differ from each other. The limited 1.8 mmHg increase in arterial to end‐tidal CO_2_ gradient as induced by a 50% increase in ${\dot{V}}_{E}/\dot{Q}$ ratio and the much smaller mismatch during LBNP provide confidence that during LBNP in the supine position the observed changes in PetCO_2_ are reflective of changes in PaCO_2_.

In summary, a relationship between CO and MCAV_mean_ beyond BP and PetCO_2_ has been demonstrated in healthy middle‐aged and elderly subjects but not in the young subjects. These data suggest that with aging, brain perfusion becomes increasingly dependent on CO irrespective of intact cerebral autoregulatory integrity.

## Conflict of Interest

None declared.
